# Long-Term Efficacy and Safety of Everolimus-Eluting Stent Implantation in Japanese Patients with Acute Coronary Syndrome: Five-Year Real-World Data from the Tokyo-MD PCI Study

**DOI:** 10.1155/2019/3146848

**Published:** 2019-11-03

**Authors:** Shunji Yoshikawa, Takashi Ashikaga, Toru Miyazaki, Ken Kurihara, Kenzo Hirao

**Affiliations:** ^1^Department of Cardiology, Tokyo Yamate Medical Center, Tokyo, Japan; ^2^Department of Cardiovascular Medicine, Tokyo Medical and Dental University, Tokyo, Japan

## Abstract

**Background:**

The long-term safety of first-generation drug-eluting stent (DES) in acute coronary syndrome (ACS) was controversial.

**Purpose:**

The purpose of this study was to establish 5-year real-world data regarding the long-term efficacy and safety of second-generation DES in Japanese patients with ACS.

**Methods:**

The Tokyo-MD PCI study is a multicenter, observational cohort study enrolling consecutive patients who underwent everolimus-eluting stent (EES) implantation. The 5-year clinical events were compared between the ACS group (*n* = 644) and the stable coronary artery disease (SCAD) group (*n* = 1255). The primary efficacy endpoint was ischemia-driven target lesion revascularization (TLR), and the primary safety endpoint was the composite of all-cause death or myocardial infarction (MI).

**Results:**

The median follow-up duration was 5.4 years. The cumulative incidence of ischemia-driven TLR was similar between ACS and SCAD (1 year: 3.0% versus 2.7%; *P*=0.682, 1–5 years: 2.7% versus 2.9%; *P*=0.864). The cumulative incidence of all-cause death or MI within 1 year was significantly higher in ACS than in SCAD (7.4% versus 3.8%; *P* < 0.001); however, ACS did not increase the risk of all-cause death or MI after adjusting confounders (adjusted hazard ratio, 1.260; 95% confidence interval, 0.774–2.053; *P*=0.352). From 1 to 5 years, the cumulative incidence of all-cause death or MI was not significantly different between ACS and SCAD (11.6% versus 11.4%; *P*=0.706). The cumulative incidence of very late stent thrombosis was low and similar between ACS and SCAD (0.2% versus 0.2%; *P*=0.942).

**Conclusion:**

This real-world registry suggested that EES has comparable long-term efficacy and safety in patients with ACS and SCAD.

## 1. Introduction

The efficacy and safety of drug-eluting stent (DES) for acute coronary syndrome (ACS) patients have been continuously discussed since it was first introduced. Several early studies showed that first-generation DES has favorable short-term efficacy for reducing target lesion revascularization (TLR) in patients with acute myocardial infarction (AMI) compared with bare metal stent (BMS) [1, 2]. However, pathological examination of first-generation DES revealed that the vessel healing of the culprit lesions of AMI patients was delayed compared with those of stable coronary artery disease (SCAD) patients [3]. A large cohort study elucidated that ACS was an independent risk factor for late stent thrombosis (ST) after first-generation DES implantation [4]. Moreover, the GRACE study reported an increased late mortality after first-generation DES implantation in patients with AMI compared with BMS [5]. In addition, prolonged dual-antiplatelet therapy (DAPT) for AMI patients was associated with the increased risk of major bleeding [6, 7].

Everolimus-eluting stent (EES) was developed to advance the safety and efficacy of DES. Target lesion failure (TLF) and ST were consistently reduced with EES compared with first-generation DES [8–10]. Guidelines for the management of ST-segment elevation myocardial infarction (STEMI) indicated new-generation DES as the first-line coronary stent for reperfusion therapy [11, 12]. Recently, the 5-year results of the EXAMINATION study demonstrated the long-term benefits of EES in AMI patients compared with BMS [13].

In Japan, EES reduced TLF compared with sirolimus-eluting stent (SES) and there was no major concern about very late ST or late catch-up phenomenon [14, 15]. However, the long-term safety of EES for Japanese ACS patients, particularly beyond 3 years, has not been fully clarified. We conducted the Tokyo MD PCI study and registered complex Japanese patient populations for evaluation of the real-world data of EES [16]. In Japan, imaging device-guided EES implantation is performed at high rates. The imaging device-guided procedure allows optimal stenting and is expected to reduce late adverse events [17]. We expected that Japan would be suitable for a multicenter observational study for the long-term evaluation of EES. Therefore, we aimed to establish 5-year real-world data regarding the long-term efficacy and safety of EES for Japanese ACS patients in this study.

## 2. Methods

### 2.1. Study Design and Enrollment of Patients

The Tokyo-MD PCI study is a physician-initiated, multicenter, observational cohort study which was conducted to evaluate the real-world data of Japanese patients who underwent EES (Xience V®; Abbott Vascular, Santa Clara, CA, USA; Promus®; Boston Scientific, Marlborough, MA, USA) implantation. Previously, we published the details and 4-year results of the Tokyo-MD PCI study [16]. From January 2010 to December 2011, consecutive 1918 patients were enrolled from 22 hospitals in Japan. The patients were divided into the ACS group or the SCAD group based on the clinical manifestation at the first procedure. The ACS group was further stratified into STEMI, non-ST elevation myocardial infarction (NSTEMI), and unstable angina pectoris (UAP) according to the Third Universal Definition of Myocardial Infarction [18]. Myocardial infarction (MI) was diagnosed when cardiac biomarker values rise above the 99^th^ percentile upper reference limit with the following: symptom of ischemia, new significant ST-T change or new left bundle branch block, development of pathological *Q* wave, imaging evidence of new regional wall motion abnormality, and identification of an intracoronary thrombus by angiography. MI accompanied with ST-segment elevation in two contiguous leads was defined as STEMI; in contrast, absence of ST-segment elevation at presentation was diagnosed as NSTEMI. Patients without elevated biomarker values were diagnosed as unstable angina.

The clinical information on patient and lesion characteristics at baseline, procedures of EES implantation, and follow-up data were retrospectively collected from medical records. Follow-up included a clinical visit or telephone contact. The median follow-up period for surviving patients was 5.4 years (interquartile range 4.3–6.1 years). Clinical follow-up was completed in 93.4% of surviving patients at 1 year, 82.3% at 3 years, and 70.7% at 5 years. Follow-up angiography within 1 year was left to the discretion of each hospital.

This study was approved by the institutional ethical review board at Tokyo Medical and Dental University and according to the Ethical Guidelines for Epidemiological Research. We published all relevant details of this study instead of obtaining informed consent.

### 2.2. Stent Implantation and Antiplatelet Therapy

The procedures of EES implantation and medical therapy, including the duration of DAPT, were left to the discretion of each attending physician. At the index procedure, heparin was used for anticoagulation. The recommended antiplatelet therapy was aspirin (81 mg/day) and thienopyridine (200 mg/day ticlopidine or 75 mg/day clopidogrel). Persistent discontinuation of DAPT was defined as withdrawal lasting 2 months.

### 2.3. Study Endpoints

The primary efficacy endpoint was ischemia-driven TLR, and the primary safety endpoint was the composite of all-cause death or MI. The secondary endpoint included cardiac death, target vessel MI, TLF (composite of cardiac death, target vessel MI, and ischemia-driven TLR), and definite and probable ST. The landmark analysis of patients who did not have the primary and secondary events at 1 year was performed. All-cause death and MI were judged by the investigator at each center. Cardiac death and MI were defined according to standardized definitions [19]. TLR was defined as either percutaneous coronary intervention (PCI) or coronary artery bypass grafting (CABG) because of restenosis or thrombosis of the target lesion, including lesions within 5 mm of the stent borders. TLR was defined as ischemia-driven if the procedure was associated with a positive functional study result or ischemic symptoms. ST was defined according to the Academic Research Consortium definition. Major bleeding event was defined as type 3 or 5 bleeding according to the Bleeding Academic Research Consortium (BARC) criteria.

### 2.4. Statistical Analysis

Categorical variables were compared using the chi-square test. Continuous variables are presented as the mean and standard deviation, and categorical variables are presented as numbers and percentages. Continuous variables were compared using Student's *t* test and Dunnett's test based on their distribution. The cumulative incidence of clinical endpoint was estimated by using the Kaplan–Meier method and compared with the log-rank test. Hazard ratio (HR) was calculated in the univariate and multivariate Cox proportional hazards models. In the univariate models, the HR for ACS and 33 confounders that were reported as being important factors in previous studies were calculated. A multivariate Cox proportional hazard model was used to adjust the differences in baseline characteristics. In the multivariable analysis, we incorporated variables with *P* values <0.05 in the univariate models. The results are expressed as adjusted HR and their 95% confidence intervals (CI). All analyses were performed using SPSS version 10 (IBM in Armonk, Cary, NY, USA).

## 3. Results

### 3.1. Patient Enrollment

**Figure 1:**
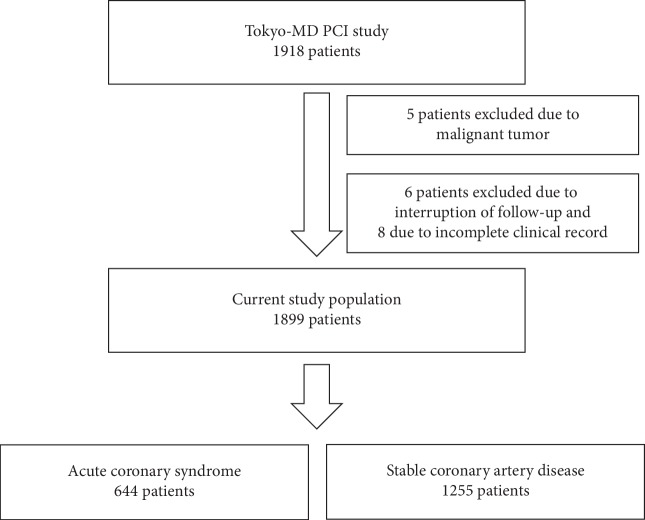
Flowchart of the Tokyo-MD PCI study.

Figure 1 shows the flowchart of the Tokyo-MD PCI study. Five patients with a malignant tumor, 6 patients who dropped out of medical follow-up, and 8 patients whose clinical records were incomplete were excluded. After the exclusion of the 19 patients, 1899 patients were evaluated. Studied patients were divided into the ACS group (*n* = 644) or the SCAD group (*n* = 1255).

### 3.2. Baseline Characteristics

Patient and lesion characteristics are shown in Table 1. The ACS patients had higher prevalence of chronic kidney disease without hemodialysis, left ventricular ejection fraction <35%, cardiogenic shock, triple-vessel disease, left anterior descending coronary artery lesion, and ostial lesion and were aged more than 80 years compared with SCAD patients. In contrast, the prevalence of diabetes mellitus, hemodialysis, peripheral artery disease, previous PCI, previous CABG, previous MI, restenotic lesion, and chronic total occlusion were higher in SCAD patients. The ACS group included patients with cardiogenic shock status (5.1%). Intra-aortic balloon pumping and percutaneous cardiopulmonary support were used in 40 (6.2%) and 10 (1.6%) patients of the ACS group. Beta-blocker was more frequently prescribed in ACS patients than in SCAD patients. An imaging device was used in 94% of the studied procedures. Follow-up coronary artery angiography within 1 year was performed in 61% of the patients.

### 3.3. DAPT Discontinuation

**Figure 2:**
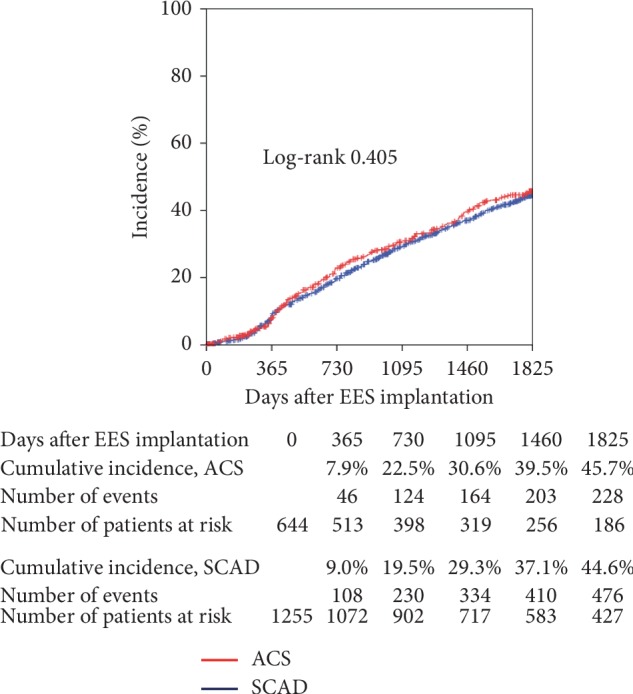
Cumulative incidence of persistent discontinuation of dual-antiplatelet therapy. Persistent discontinuation was defined as withdrawal lasting for at least 2 months. EES: everolimus-eluting stent; ACS: acute coronary syndrome; SCAD: stable coronary artery disease.

Figure 2 shows the cumulative incidence of persistent discontinuation of DAPT. DAPT was discontinued in 7.9% at 1 year and in 45.7% at 5 years in the ACS group and in 9.0% at 1 year and in 44.6% at 5 years in the SCAD group. There was no significant difference in persistent discontinuation of DAPT at 5 years between the two groups (*P*=0.405).

### 3.4. Clinical Outcome

**Figure 3:**
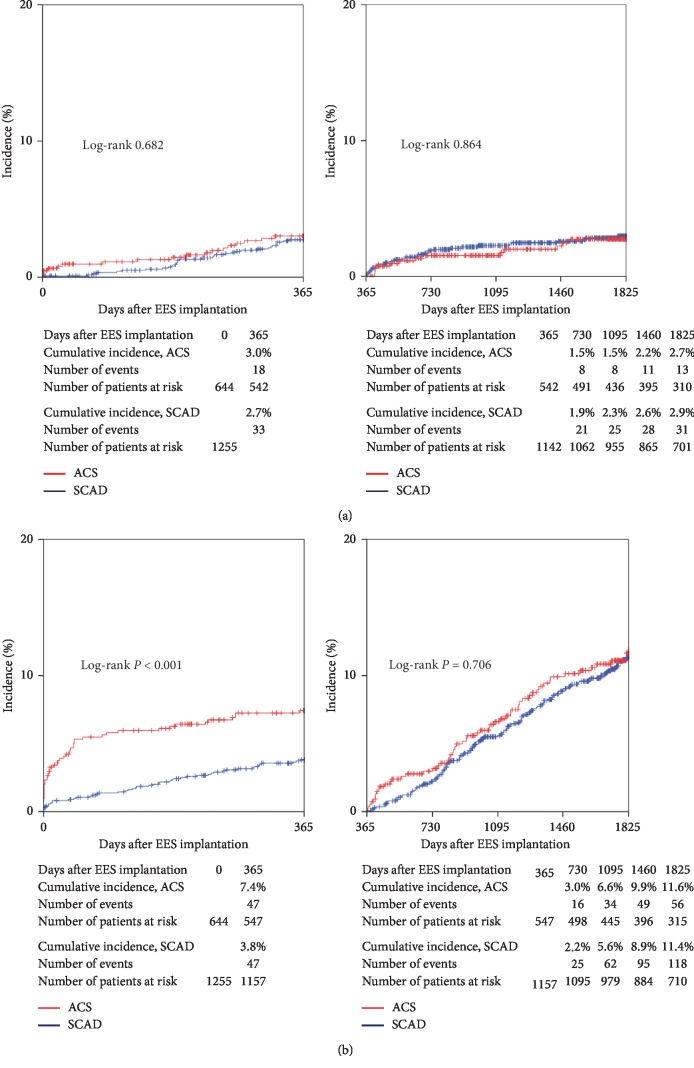
(a) Cumulative incidence of the primary efficacy endpoint (ischemia-driven target lesion revascularization). (b) Cumulative incidence of the primary safety endpoint (the composite of all-cause death or myocardial infarction). EES: everolimus-eluting stent; ACS: acute coronary syndrome; SCAD: stable coronary artery disease.

Table 2 shows the clinical events in the ACS and SCAD groups.  Figure 3(a) shows the cumulative incidence of the primary efficacy endpoint (ischemia-driven TLR). The cumulative incidence of ischemia-driven TLR was similar between the ACS and SCAD groups at 5 years (1 year: 3.0% versus 2.7%; *P*=0.682; from 1 to 5 years: 2.7% versus 2.9%; *P*=0.864).

The cumulative incidence of the safety endpoint (the composite of all-cause death or MI) is shown in Figure 3(b). The cumulative incidence of all-cause death or MI at 1 year was significantly higher in the ACS group than in the SCAD group (7.4% versus 3.8%; *P* < 0.001). The higher risk of the safety endpoint in the ACS group at 1 year was mainly driven by the increase in all-cause death compared with the SCAD group (5.5% versus 2.4%; *P* < 0.001). From 1 to 5 years, the cumulative incidence of all-cause death or MI was not significantly different between the ACS and SCAD groups (11.6% versus 11.4%; *P*=0.706).

**Figure 4:**
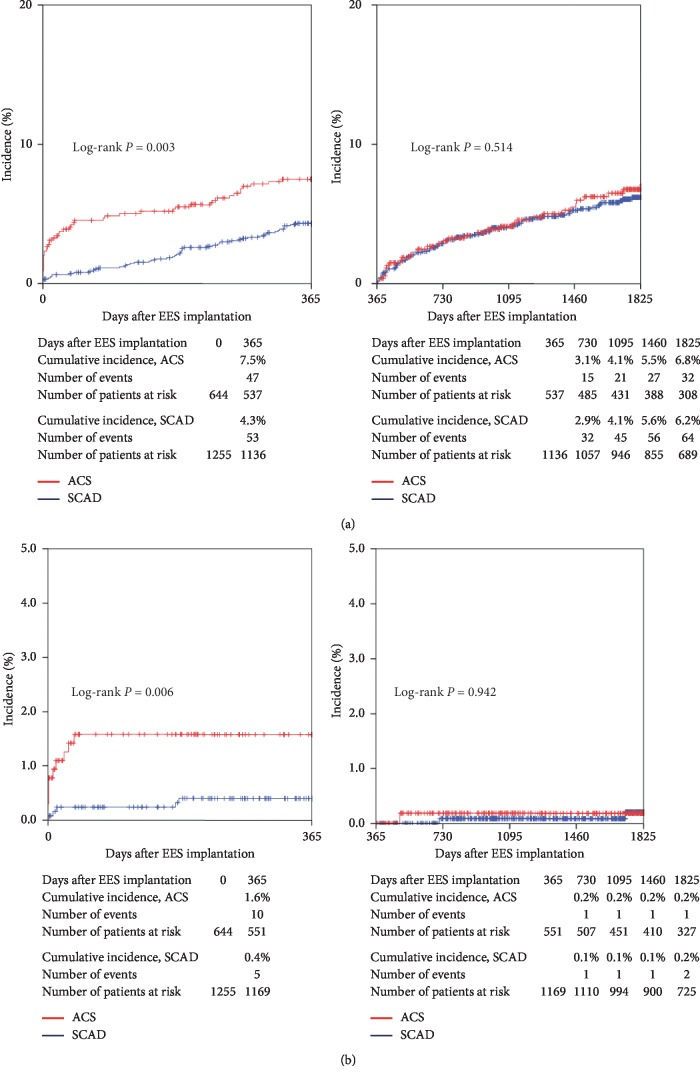
(a) Cumulative incidence of target lesion failure. (b) Cumulative incidence of stent thrombosis. EES: everolimus-eluting stent; ACS: acute coronary syndrome; SCAD: stable coronary artery disease.

Figure 4(a) shows the cumulative incidence of TLF. TLF occurred significantly more frequently in the ACS group than in the SCAD group at 1 year (7.5% versus 4.3%; *P*=0.003). From 1 to 5 years, the cumulative incidence of TLF was not significantly different between the ACS and SCAD groups (6.8% versus 6.2%; *P*=0.514).

Figure 4(b) shows the cumulative incidence of definite and probable ST. ST within 1 year was observed significantly more frequent in the ACS group than in the SCAD group (1.6% versus 0.4%; *P*=0.006). However, the incidence of ST from 1 to 5 years was very low in both groups, and there was no significant difference between the ACS and SCAD groups (0.2% versus 0.2%; *P*=0.942).

### 3.5. Univariate and Multivariate Analysis of the Primary Safety End-point

We performed the univariate and multivariate analysis to calculate the adjusted HR of the primary safety endpoint (Table 3 and 4) because there was significantly increased risk in the safety endpoint in the ACS group at 1 year.

At 1 year, the univariate analysis showed that the ACS group had higher HR of the safety endpoint compared with the SCAD group (HR, 2.033; 95% CI, 1.357–3.046; *P*=0.001). The multivariate analysis revealed that age older than 80 years, hemodialysis, left ventricular ejection fraction <35%, cardiogenic shock, and ostial lesion had higher adjusted HR of the safety endpoint at 1 year. However, the adjusted HR of the ACS group at 1 year was not significantly higher compared with the SCAD group (adjusted HR, 1.260; 95% CI, 0.774–2.053; *P*=0.352).

From 1 to 5 years, the ACS group did not increase the HR of the safety endpoint compared with the SCAD group (HR, 1.058; 95% CI, 0.706–1.418; *P*=0.70). By the multivariate analysis, age older than 80 years, chronic kidney disease without hemodialysis, hemodialysis, left ventricular ejection fraction <35%, peripheral artery disease, and anticoagulation therapy were determined as independent predictors of the safety endpoint from 1 to 5 years.

## 4. Discussion

In the present study, we have for the first time compared the 5-year real-world outcome after EES implantation between ACS and SCAD patients in Japan. The main findings are as follows. (1) The incidence of efficacy endpoint was similar between the two groups at 5 years. (2) The incidence of safety endpoint and TLF within 1 year was significantly higher in the ACS group compared with the SCAD group. However, those were not different beyond 1 year. The multivariate analysis clarified that ACS did not increase the adjusted HR of the safety endpoint throughout 5 years. (3) The cumulative incidence of ST beyond 1 year was very low and similar between ACS and SCAD groups.

ACS has common pathological backgrounds, including disruption or erosion of the atherosclerotic plaques, alterations in circulating prothrombotic or antifibrinolytic mediators, and acute coronary thrombogenicity [20]. Although ACS treatment has notably developed, STEMI still has approximately 5 to 6% in-hospital mortality and 7 to 18% 1-year mortality rates [11].

Previously, the long-term outcome of Japanese ACS patients treated with first-generation DES was reported in the largest SES study (j-Cypher) and in the single-center registry of Kyoto University Hospital (which used SES in 82% of the patients) [21, 22]. Although the Tokyo-MD PCI study was retrospective, we performed consecutive patient registrations from multicenter hospitals and there were small numbers of excluded patients.

The Tokyo-MD PCI study had several remarkable differences compared with previous overseas studies of EES on the following points. (1) Compared with the XIENCE V USA study (an all-comer observational registry in the United States) [23], the Tokyo-MD PCI study registered more complex patient populations, such as those undergoing hemodialysis, with cardiogenic shock status, with low ejection fraction, with left main disease, and with angiographic heavy calcification. (2) The rate of imaging device-guided EES implantation was high. The imaging device-guided procedure is expected to reduce late adverse events. These features of this study distinguished the Tokyo-MD PCI study from previous EES studies. Therefore, we think this registry has the new information about the clinical practice of EES implantation.

### 4.1. Problems of First-Generation DES Implantation in ACS Patients

First-generation DES has the problem of late catch-up phenomenon and late ST [24, 25]. Clinical researches examining the safety of first-generation DES in ACS patients showed conflicting results about the risk of death or ST [5, 26–29]. The pathology of first-generation DES revealed the presence of delayed arterial healing [30], and incomplete endothelial coverage was the histological predictor of ST [31]. Regarding the culprit lesions of ACS, underlying plaque morphology including large necrotic core, ruptured fibrous cap, and thrombus attributed to the further delayed arterial response to first-generation DES [3]. Optical coherence tomography (OCT) also validated the further delayed vascular healing in patients with UAP after SES implantation compared with SCAD [32]. This distinctive response to first-generation DES between ACS and SCAD lesions implies that lesion morphology plays an important role in vascular healing. Moreover, heterogeneous healing responses remained after 5 years of first-generation DES implantation [33].

### 4.2. Improved Vascular Response of EES Compared with First-Generation DES

EES consists of a thin strut platform (81 *μ*m), coated with 7.8-*μ*m-thick durable fluorinated copolymer and 1.0 *μ*g/mm everolimus [34]. In human autopsy analysis, EES showed favorable strut coverage with less inflammation and fibrin deposition compared with SES and paclitaxel-eluting stent (PES) [35]. Sawada compared the arterial healings in STEMI patients at 7 months between EES and SES using OCT and angioscopy [36]. OCT showed that frequencies of uncovered and malapposed struts in EES were lower than those in SES in STEMI patients. Angioscopic analysis also presented more homogenous neointimal coverage and less intrastent thrombus in EES than those in SES. These improved pathological findings support the greater clinical safety of EES compared with first-generation DES in ACS patients.

### 4.3. Efficacy Endpoint after EES Implantation in ACS Patients

In the Tokyo-MD PCI study, EES had comparable efficacy in reducing ischemia-driven TLR between the ACS and SCAD groups at 5 years. Similar result was previously observed in the j-Cypher study, in which the incidence of TLR after SES implantation was not different between the ACS and non-ACS patients at 3 years [21]. In the XIENCE V USA study, AMI was not associated with the increased risk of TLR [23]. Our data conformed to the existing data showing that ACS did not increase the risk of TLR after DES implantation.

### 4.4. Safety Endpoint after EES Implantation in ACS Patients

In the Tokyo-MD PCI study, the ACS group had the higher risk of the safety endpoint at 1 year than in the SCAD group. However, multivariate analysis revealed that ACS was not an independent predictor of the safety endpoint at 1 year. This study demonstrated that severity and comorbidity of ACS patients, such as hemodialysis, low ejection fraction, and cardiogenic shock status, were mainly attributed to the risk of the safety endpoint at 1 year.

In contrast, the incidence of the safety endpoint from 1 to 5 years was similar between the ACS and SCAD groups despite the differences in patient background. Although the incidence of MI was more frequent in the ACS group than in the SCAD group from 1 to 5 years, the number of MI events was small in both groups, resulting in no statistical difference in the safety endpoint. In addition, the low incidence of very late ST in both groups might contribute to the favorable long-term safety after EES implantation.

### 4.5. ST after EES Implantation in Patients with ACS

The cumulative incidence of ST at 1 year was higher in the ACS group than in the SCAD group in the Tokyo-MD PCI study, and most incidence of ST occurred within 1 month. These results were explained because stenting for the culprit lesions of ACS has higher risk of acute and subacute ST due to the instability of the atheromatous plaque and the presence of thrombus [37].

The incidence of ST beyond 1 year was low and not different between the two groups. In the era of first-generation DES, subanalysis of the j-Cypher study showed that ACS patients tend to have higher risk of ST beyond 1 year than non-ACS patients, and ST continued to occur up to 3 years in ACS patients [21]. In the Kyoto University registry, definite ST occurred in 3.0% of ACS patients at 5 years after DES implantation in Japan [22].

EES reduced the risk of very late ST in ACS patients compared with first-generation DES in the subgroup analysis of a prospective cohort study [38]. In addition, the Tokyo-MD PCI study had similar incidence of definite or probable ST at 5 years compared with the EXAMINATION study (Tokyo-MD PCI 1.8% versus EXAMINATION 2.0%) [13]. Our data were consistent with those of previous overseas studies demonstrating the benefit of EES in reducing the risk of late ST in ACS patients.

However, the incidence of ST in the Tokyo-MD PCI study was too low to analyze the differences between the two groups and to decide the predictors of ST by the multivariate analysis. The evolution of new-generation DES substantially reduced late and very late ST; therefore, a larger-scale clinical study is required for more detailed statistical analysis.

### 4.6. Unsolved Problems of EES

Another pathological problem of DES was lipid-rich neoatherosclerosis, which might lead to subsequent ST from the disruption of neointimal hyperplasia. A previous study reported the frequencies of neoatherosclerosis were similar between EES and SES [36]. It is uncertain whether ACS increases the risk of neoatherosclerosis after EES implantation.

The appropriate DAPT duration after DES implantation has not been established yet. The guideline on the duration of DAPT recommended 6 months DAPT for SCAD and at least 12 months DAPT for ACS [39]. However, the duration of DAPT in this study was longer than that of the current guideline in both groups. In the early period of EES implantation, physicians preferred the longer DAPT duration for the concern of the late ST. Recently, several clinical studies have tried to shorten the duration of DAPT [40]. Further studies are needed to examine whether the duration of DAPT affects clinical outcome in ACS patients.

The rate of imaging device use was high in the Tokyo-MD PCI study. The univariate analysis showed that the use of imaging device reduced the risk of safety endpoint within 1 year compared with the nonuse of imaging device. The recent study showed that intravascular ultrasound-guided primary PCI for STEMI was not associated with a lower risk for target-vessel revascularization or ST [41]. The efficacy of imaging devices in DES implantation for ACS patients should be investigated further.

### 4.7. Study Limitation

This study has several limitations. First, this registry was a nonrandomized, observational cohort study. There was a possibility that unknown confounding factors had influence on the clinical events. Second, clinical information was collected retrospectively. Although the median follow-up period exceeded 5 years, under-reporting of the clinical events was possible. Third, the procedure of EES implantation and the duration of DAPT were left to the discretion of each attending physician. Detailed information on procedures, such as thrombus aspiration, distal protection, and direct stenting was not available. Consequently, this study did not allow the analysis of the effects of the procedure and the medical therapy in the occurrences of the clinical endpoints. Forth, this study is the subanalysis of the Tokyo-MD PCI study and we do not have sample size estimation. Fifth, the number of studied patients was small for the statistical analysis of the risk of ST. Further studies are needed to investigate the risk of very late ST in ACS patients.

## 5. Conclusion

This real-world registry suggested that EES has comparable long-term efficacy and safety in Japanese patients with ACS and SCAD at 5-year follow-up. These findings might have a great impact on determining the strategy of revascularization therapy for ACS patients.

## Figures and Tables

**Table 1:** Patient and lesion characteristics.

Variable	ACS group	SCAD group	*P* value
Patients (*n*)	644	1255	
Age (years)	70.5 ± 9.9	69.6 ± 9.9	0.050
Age ≥80 years	135 (21.0%)	195 (15.5%)	0.003
Male	461 (71.6%)	945 (75.3%)	0.080
ST-segment elevation myocardial infarction	218 (33.9%)	0	<0.001
Non-ST-segment elevation myocardial infarction	190 (29.5%)	0	<0.001
Unstable angina pectoris	236 (36.6%)	0	<0.001
Stable angina pectoris	0	885 (70.1%)	<0.001
Silent myocardial ischemia	0	370 (29.5%)	<0.001
Current smoker	139 (20.7%)	233 (18.6%)	0.275
Hypertension	469 (72.8%)	922 (73.5%)	0.765
Dyslipidemia	533 (82.8%)	1025 (81.7%)	0.558
Diabetes mellitus	244 (37.9%)	544 (43.3%)	0.022
Chronic kidney disease			
Without hemodialysis	129 (20.0%)	200 (15.9%)	0.027
With hemodialysis	24 (3.7%)	102 (8.1%)	<0.001
Cardiogenic shock state at procedure	33 (5.1%)	0	<0.001
Intra-aortic balloon pumping	40 (6.2%)	3 (0.2)	<0.001
Percutaneous cardiopulmonary support	10 (1.6%)	0 (0.0)	<0.001
Left ventricular ejection fraction <35%	48 (7.5%)	57 (4.5%)	0.009
Triple vessel disease	106 (16.5%)	155 (12.4%)	0.014
Peripheral artery disease	50 (7.8%)	140 (11.2%)	0.020
History of stroke	59 (9.2%)	129 (10.3%)	0.440
History of myocardial infarction	156 (24.2%)	419 (33.4%)	<0.001
Previous percutaneous coronary intervention	172 (26.7%)	509 (40.6%)	<0.001
Previous coronary artery bypass grafting	30 (4.7%)	89 (7.1%)	0.038
Medication			
Use of aspirin	641 (99.5%)	1251 (99.7%)	0.617
Use of thienopyridine	636 (98.8%)	1229 (97.9%)	0.197
Use of cilostazol	20 (3.1%)	45 (3.6%)	0.586
Use of anticoagulant agent	69 (10.7%)	143 (11.4%)	0.656
Use of statin	509 (79.0%)	985 (78.5%)	0.781
Use of beta-blocker	348 (54.0%)	562 (44.8%)	<0.001
Use of angiotensin-converting enzyme inhibitor	107 (16.6%)	173 (13.8%)	0.100
Use of angiotensin receptor blocker	282 (43.8%)	566 (45.1%)	0.586
Use of proton pump inhibitor	387 (60.1%)	752 (59.9%)	0.942
Coronary lesion location			<0.001
Left anterior descending coronary artery	311 (48.3%)	519 (41.4%)	
Left circumflex coronary artery	100 (15.5%)	246 (19.6%)	
Right coronary artery	184 (28.6%)	435 (34.7%)	
Left main coronary artery	47 (7.3%)	51 (4.1%)	
Bypass graft	2 (0.3%)	4 (0.3%)	
Type B2/C lesion	509 (79.0%)	982 (78.2%)	0.691
Restenotic lesion	56 (8.7%)	151 (12.0%)	0.027
Chronic total occlusion	24 (3.7%)	121 (9.6%)	<0.001
Ostial lesion	104 (16.1%)	169 (13.5%)	0.115
Bifurcation	160 (24.8%)	301 (24.0%)	0.679
Severe calcification on angiography	130 (20.2%)	275 (21.9%)	0.385
Number of stents used (*n*)	1.4 ± 0.7	1.4 ± 0.7	0.742
Average stent diameter (mm)	2.98 ± 0.36	2.97 ± 0.36	0.626
Use of 2.5 mm diameter stent	212 (32.9%)	439 (35.0%)	0.370
Total stent length (mm)	29.5 ± 17.4	29.1 ± 17.4	0.737
Total stent length ≥28 mm	193 (30.0%)	375 (29.9%)	0.968
Use of imaging device for stent placement	565 (87.7%)	1224 (97.5%)	<0.001
Initial thrombolysis in myocardial infarction grade flow			<0.001
0	131 (20.3%)	121 (9.6%)	
1	49 (1.6%)	9 (0.7%)	
2	105 (16.3%)	12 (0.9%)	
3	359 (55.7%)	1113 (88.7%)	
Final thrombolysis in myocardial infarction grade flow			0.149
0	1 (0.2%)	0	
1	5 (0.8%)	3 (0.2%)	
2	10 (1.6%)	15 (1.2%)	
3	628 (97.6%)	1237 (98.6%)	

*Note*. Data are given as *n* (%) or as the mean ± standard deviation. ACS: acute coronary syndrome; SCAD: stable coronary artery disease.

**Table 2:** Clinical events through 5 years.

	At 1 year			From 1 to 5 years		
	ACS	SCAD	*P* value	ACS	SCAD	*P* value
All-cause death	35 (5.5%)	30 (2.4%)	<0.001	44 (9.1%)	105 (10.0%)	0.460
Cardiac death	25 (3.9%)	15 (1.2%)	<0.001	18 (3.7%)	32 (3.1%)	0.373
Myocardial infarction	15 (2.4%)	18 (1.5%)	0.132	16 (3.3%)	17 (1.8%)	0.008
Target vessel myocardial infarction	11 (1.8%)	9 (0.7%)	0.039	7 (1.4%)	10 (1.0%)	0.227
All-cause death or myocardial infarction	47 (7.4%)	47 (3.8%)	<0.001	56 (11.6%)	118 (11.4%)	0.706
Ischemia-driven TLR	18 (3.0%)	33 (2.7%)	0.682	13 (2.7%)	31 (2.9%)	0.864
Target lesion failure	47 (7.5%)	53 (4.3%)	0.003	32 (6.8%)	64 (6.2%)	0.514
BARC bleeding						
Type 3	8 (1.3%)	12 (1.0%)	0.498	7 (1.6%)	26 (2.6%)	0.085
Type 5	0 (0.0%)	3 (0.2%)	0.229	2 (0.4%)	3 (0.3%)	0.705
Stent thrombosis						
Definite	8 (1.3%)	4 (0.3%)	0.015	1 (0.2%)	2 (0.2%)	0.942
Probable	2 (0.3%)	1 (0.1%)	0.223	0 (0%)	0 (0%)	NA
Definite or probable	10 (1.6%)	5 (0.4%)	0.006	1 (0.2%)	2 (0.2%)	0.942
Possible	2 (0.3%)	5 (0.4%)	0.814	15 (1.5%)	8 (1.6%)	0.974

*Note*. Incidences of events were calculated by the Kaplan–Meyer method. Cumulative incidences were compared with the log-rank test. ACS: acute coronary syndrome; SCAD: stable coronary artery disease; TLR: target lesion revascularization; BARC: bleeding academic research consortium.

**Table 3:** Univariate and multivariate analysis for the safety endpoint (the composite of all-cause death or myocardial infarction) at 1 year.

	Univariate Cox proportional hazard model		Multivariate Cox proportional hazard model	
	HR (95% CI)	*P* value	HR (95% CI)	*P* value
ACS versus SCAD	2.033 (1.357–3.046)	0.001	1.260 (0.774–2.053)	0.352
Age ≥80 years	2.369 (1.536–3.656)	<0.001	2.145 (1.358–3.386)	0.001
Male	0.732 (0.475–1.130)	0.159		
Current smoker	1.260 (0.782–2.032)	0.342		
Hypertension	1.201 (0.745–1.937)	0.451		
Dyslipidemia	0.459 (0.298–0.708)	<0.001	0.993 (0.486–2.029)	0.985
Diabetes mellitus	1.291 (0.861–1.935)	0.216		
Chronic kidney disease without hemodialysis	1.484 (0.921–2.393)	0.105		
Chronic kidney disease with hemodialysis	4.658 (2.927–7.499)	<0.001	3.895 (2.124–7.143)	<0.001
Left ventricular ejection fraction <35%	8.352 (5.366–13.002)	<0.001	3.223 (1.913–5.431)	<0.001
Peripheral artery disease	2.009 (1.118–3.396)	0.009	1.261 (0.692–2.299)	0.449
History of stroke	1.608 (0.911–2.837)	0.101		
History of myocardial infarction	0.923 (0.590–1.442)	0.724		
Previous percutaneous coronary intervention	0.825 (0.535–1.273)	0.384		
Previous coronary artery bypass grafting	1.403 (0.680–2.895)	0.360		
Triple vessel disease	2.470 (1.527–3.881)	<0.001	1.380 (0.844–2.255)	0.199
Cardiogenic shock status at procedure	28.050 (16.864–46.656)	<0.001	14.228 (6.511–31.090)	<0.001
Left anterior descending coronary artery	0.835 (0.552–1.263)	0.392		
Left circumflex coronary artery	0.584 (0.311–1.095)	0.094		
Right coronary artery	1.345 (0.889–2.034)	0.161		
Left main coronary artery	2.066 (1.039–4.108)	0.038	1.360 (0.560–1.807)	0.360
Type B2/C lesion	1.677 (0.933–3.011)	0.084		
Restenotic lesion	0.546 (0.239–1.247)	0.151		
Chronic total occlusion	1.119 (0.542–2.309)	0.761		
Ostial lesion	2.921 (1.893–4.506)	<0.001	2.605 (1.600–4.239)	<0.001
Bifurcation	1.349 (0.867–2.099)	0.185		
Severe calcification on angiography	2.725 (1.808–4.107)	<0.001	1.364 (0.845–2.203)	0.204
Use of 2.5 mm diameter stent	1.378 (0.914–2.077)	0.126		
Total stent length ≥28 mm	1.479 (0.976–2.242)	0.065		
Use of imaging device for stent placement	0.281 (0.164–0.481)	<0.001	0.765 (0.378–1.547)	0.456
Use of anticoagulant agent	0.937 (0.499–1.757)	0.838		
Use of statin	0.429 (0.283–0.652)	<0.001	0.853 (0.419–1.737)	0.662
Use of beta-blocker	0.752 (0.499–1.133)	0.173		
Use of angiotensin-converting enzyme inhibitor	0.625 (0.408–0.957)	0.031	0.684 (0.436–1.073)	0.098
Use of angiotensin receptor blocker	1.176 (0.687–2.014)	0.554		

*Note*. ACS: acute coronary syndrome; SCAD: stable coronary artery disease; HR: hazard ratio; CI: confidence interval.

**Table 4:** Univariate and multivariate analysis for the safety endpoint (the composite of all-cause death or myocardial infarction) from 1 to 5 years.

	Univariate Cox proportional hazard model		Multivariate Cox proportional hazard model	
	HR (95% CI)	*P* value	HR (95% CI)	*P* value
ACS versus SCAD	1.058 (0.789–1.418)	0.706		
Age ≥80 years	2.192 (1.606–2.992)	<0.001	2.079 (1.511–2.860)	<0.001
Male	1.099 (0.793–1.522)	0.571		
Current smoker	0.735 (0.501–1.077)	0.114		
Hypertension	1.283 (0.922–1.786)	0.140		
Dyslipidemia	0.615 (0.447–0.846)	0.003	0.749 (0.456–1.231)	0.254
Diabetes mellitus	1.189 (0.903–1.564)	0.217		
Chronic kidney disease without hemodialysis	1.931 (1.412–2.641)	<0.001	1.747 (1.244–2.454)	0.001
Chronic kidney disease with hemodialysis	3.966 (2.731–5.759)	<0.001	3.000 (1.931–4.661)	<0.001
Left ventricular ejection fraction <35%	2.931 (1.866–4.604)	<0.001	2.158 (1.345–3.464)	<0.001
Peripheral artery disease	2.663 (1.897–3.739)	<0.001	1.992 (1.384–2.868)	<0.001
History of stroke	1.645 (1.110–2.473)	0.013	1.221 (0.815–1.831)	0.333
History of myocardial infarction	1.307 (0.982–1.739)	0.067		
Previous percutaneous coronary intervention	1.150 (0.869–1.521)	0.329		
Previous coronary artery bypass grafting	1.595 (1.005–2.530)	0.047	0.921 (0.567–1.497)	0.741
Triple vessel disease	1.625 (1.146–2.303)	0.006	1.252 (0.873–1.797)	0.222
Cardiogenic shock status at procedure	4.032 (1.287–12.625)	0.017	2.984 (0.909–9.792)	0.071
Left anterior descending coronary artery	0.742 (0.559–0.985)	0.039	0.777 (0.580–1.041)	0.091
Left circumflex coronary artery	1.175 (0.839–1.645)	0.347		
Right coronary artery	1.175 (0.883–1.562)	0.268		
Left main coronary artery	1.221 (0.665–2.242)	0.519		
Type B2/C lesion	1.622 (1.112–2.365)	0.012	1.444 (0.983–2.121)	0.061
Restenotic lesion	1.095 (0.715–1.676)	0.677		
Chronic total occlusion	0.742 (0.423–1.301)	0.297		
Ostial lesion	1.139 (0.772–1.678)	0.510		
Bifurcation	0.952 (0.688–1.319)	0.769		
Severe calcification on angiography	1.888 (1.401–2.544)	<0.001	1.183 (0.852–1.642)	0.315
Use of 2.5 mm diameter stent	0.865 (0.644–1.163)	0.337		
Total stent length ≥28 mm	1.096 (0.812–1.478)	0.550		
Use of imaging device for stent placement	1.064 (0.564–2.009)	0.848		
Use of anticoagulant agent	1.843 (1.309–2.595)	<0.001	1.428 (1.003–2.035)	0.048
Use of statin	0.636 (0.467–0.866)	0.004	0.946 (0.578–1.549)	0.826
Use of beta-blocker	1.038 (0.789–1.365)	0.791		
Use of angiotensin-converting enzyme inhibitor	0.903 (0.685–1.190)	0.470		
Use of angiotensin receptor blocker	1.084 (0.739–1.588)	0.681		

*Note*. ACS: acute coronary syndrome; SCAD: stable coronary artery disease; HR: hazard ratio; CI: confidence interval.

## Data Availability

The data used to support the findings of this study are restricted by the ethical review board at Tokyo Medical and Dental University in order to protect patient's privacy. Data are available from Shunji Yoshikawa for researchers who meet the criteria for access to confidential data.

## Supplementary Materials

Supplementary Materials Participating centers and investigators.
