# Supplementary material for: Long-Term Efficacy and Safety of Everolimus-Eluting Stent Implantation in Japanese Patients with Acute Coronary Syndrome: Five-Year Real-World Data from the Tokyo-MD PCI Study
Source: J Interv Cardiol. 2019 Nov 3;2019:3146848. doi: 10.1155/2019/3146848 (PMC6874987; doi:10.1155/2019/3146848)
Supplement: Supplementary Materials — Participating centers and investigators. [file 3146848.f1.pdf]

The participating centers and investigators

Kazuo Kobayashi, Hirokazu Ohigashi, Koji Sugiyama, Kashiwa Municipal Hospital

Akihiko Matsumura, Kameda Medical Center

Shigeo Shimizu, Masakazu Ohno, National Disaster Medical Center

Hidenobu Takei, Sanraku Hospital

Tsuyoshi Tokunaga, Eijiro Hattori, JA Toride Medical Center

Michio Usui, Tokyo Yamate Medical Center

Ryo Goto, Shuuwa Medical Hospital

Tsunekazu Kakuta, Tsuchiura Kyodo General Hospital

Hiromasa Adachi, Hiroaki Yamaguchi, Yoichi Ohtaki, Tokyo Kyosai Hospital

Akihiro Hata, Tatsuya Fujinami, Toshima Hospital

Nobuhisa Obuchi, Tokyo Metropolitan Otsuka Hospital

Yasuhiro Sato, Yuko Onishi, Hiratsuka Kyosai Hospital

Takanobu Miyamoto, Nozato Toshihiro, Musashino Red Cross Hospital

Hiroyuki Hikita, Yokosuka Kyosai Hospital

Manabu Kurabayashi, Mitsutoshi Asano, Yokohama City Minato Red Cross Hospital

Hiroyuki Fujii, Masato Shimizu, Shigeki Kimura Yokohama Minami Kyosai Hospital

Atsuyuki Ohno, Kasai Shoikai Hospital

Kaoru Sakurai, Yuji Konishi, Shin-Yurigaoka general hospital
